# Public health round-up

**DOI:** 10.2471/BLT.24.010224

**Published:** 2024-02-01

**Aid being blocked**

A mother and child flee on foot from Wad Madani, Al-Gezira state, Sudan, following the outbreak of hostilities there in December 2023. Sudan is one of several countries and territories where humanitarian aid is being deliberately blocked or safety concerns are preventing its delivery. As of 10 January, the World Health Organization (WHO) had temporarily halted operations at its operational hub in Al-Gezira state.

**Figure:**
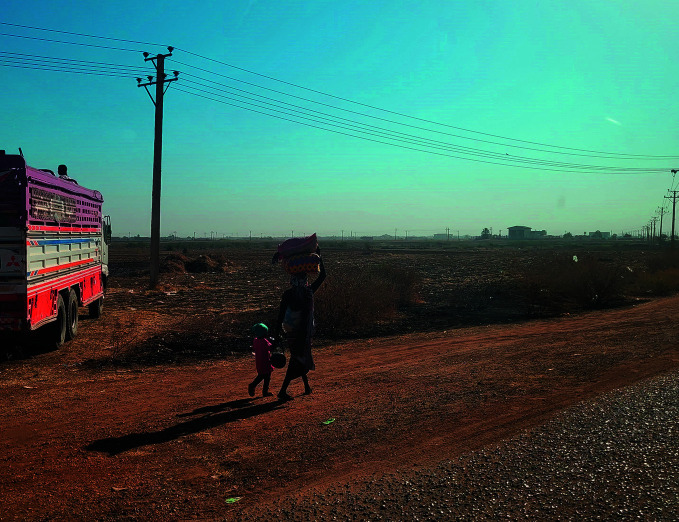
UNICEF/ Ahmed Elfatih Mohamdeen

## Hampering humanitarian aid

2024 began with several countries and territories struggling to access urgently needed humanitarian aid.

In a 10 January media briefing, World Health Organization (WHO) Director-General, Tedros Adhanom Ghebreyesus, reported that WHO had been obliged to cancel seven planned missions to northern Gaza between 26 December 2023 and 10 January 2024 because requests for access to the enclave had been rejected or assurances of safe passage had not been provided.

Some 1.9 million people – almost 90% of the Gaza population – have been displaced, many having been forced to move multiple times; access to food and water has been severely curtailed and only 15 hospitals are functioning even partially. Lack of clean water and sanitation, and overcrowded living conditions were creating the ideal environment for outbreaks of diseases.

The Director-General called on Israel to approve requests by WHO and other partners to deliver humanitarian aid, stating that even without a ceasefire, corridors could be established to allow the safe passage of humanitarian aid and workers. He also called on all sides to protect healthcare, in accordance with their obligations under international humanitarian law, stressing that healthcare cannot be attacked or militarized.

The Director-General also drew attention to Sudan, where the situation is continuing to deteriorate after nine months of conflict. Here too, the work of WHO and its partners is being hampered by increasing violence, insecurity and looting. Due to security concerns, WHO temporarily halted its operations in Al-Gezira state, which used to be a safe haven from the conflict in Khartoum and is a hub for WHO’s operations.

In Ethiopia the delivery of aid is being hampered due to an internet blackout which is severely impeding communication between health partners and authorities and restrictions on movement which is hampering the delivery of humanitarian assistance.

In related news, on 15 January, WHO launched an appeal for 1.5 billion United States dollars to protect the health of the most vulnerable people in 41 emergencies globally. The appeal aims to reach over 87 million people and is being issued in a context of complex emergencies cutting across crises of conflict, climate change and economic instability.

https://bit.ly/48N6cSQ

## WHO prequalifies second malaria vaccine

WHO announced its prequalification of the R21/Matrix-M malaria vaccine on 21 December 2023. The R21 vaccine is the second malaria vaccine prequalified by WHO, following the RTS,S/AS01 vaccine which was prequalified in July 2022. Both vaccines have been shown to be safe and effective in clinical trials, for preventing malaria in children.

Prequalification is a prerequisite for vaccine procurement by the United Nations Children’s Fund (UNICEF) and for funding support for deployment by Gavi, the Vaccine Alliance.

To date, the supply of malaria vaccines has fallen short of demand. The availability of two WHO-recommended, prequalified malaria vaccines is expected to change that.

https://bit.ly/3HeChY3

## Addition to neglected tropical disease list

WHO announced the inclusion of noma (cancrum oris or gangrenous stomatitis) in its official list of neglected tropical diseases. Announced 15 December 2023, the decision was recommended at the 17th meeting of the Strategic and Technical Advisory Group for Neglected Tropical Diseases, and underscores WHO’s commitment to expanding health services to the world's most vulnerable populations.

A severe gangrenous disease of the mouth and face, noma primarily affects malnourished children between the ages of 2 and 6 years living in extreme poverty. It starts as an inflammation of the gums, which, if not treated early, spreads quickly to destroy facial tissues and bones. It frequently leads to death, with survivors suffering severe disfigurement. Most cases are found in sub-Saharan Africa. Estimating prevalence is hampered by low levels of reporting.

https://bit.ly/48RAnbk

## COVAX closes

The COVID-19 Vaccines Global Access (COVAX) initiative wound up operations on 31 December. Launched in 2020, COVAX served as a multilateral mechanism for equitable global access to coronavirus disease 2019 (COVID-19) vaccines and was jointly led by the Coalition for Epidemic Preparedness Innovations, Gavi, UNICEF and WHO.

COVAX supplied nearly 2 billion COVID-19 vaccine doses and safe injection devices to 146 economies and is estimated to have helped avert the deaths of at least 2.7 million people.

The lower-income economies that were eligible to participate in the programme with support from the financing mechanism known as the Gavi COVAX Advance Market Commitment will continue to have the option to receive COVID-19 vaccine doses and delivery support through Gavi’s regular programmes.

As of 19 December, 58 lower-income economies had requested a total of 83 million doses in 2024, with plans to focus on the continued protection of priority groups, including health care workers, community workers and older adults. COVID-19 continues to be a concern, as noted by the Director General in the 10 January media briefing referenced above.

https://bit.ly/3NZk58q

## Polio emergency maintained

The thirty-seventh meeting of the Emergency Committee under the *International health regulations* on the international spread of poliovirus ended with unanimous agreement that the risk of international spread of the virus remains a Public Health Emergency of International Concern (PHEIC).

In a 22 December statement the committee recommended the PHEIC’s extension for a further three months in view of the ongoing risk of wild and vaccine-derived poliovirus strain transmission and polio response concerns that include weak routine immunization in many countries (notably those impacted by humanitarian emergencies) and lack of access to vaccine particularly in northern Yemen and Somalia which have sizable populations that have not been vaccinated for more than a year.

https://bit.ly/3SfXGpY

## Cholera spread in 2023

Data from Member States indicate that the number of cholera cases reported in 2023 surpassed that of 2022, although changes in surveillance systems and capacity make direct comparison impossible.

According to a WHO situation report issued on 11 January 2024, there were 667 000 cases and 4000 deaths in 2023, with 30 countries reporting cases since the beginning of the year. In 2022, 472 697 cholera cases were reported to WHO.

WHO classified the global resurgence of cholera as a grade 3 emergency (the highest internal level for a health emergency requiring a comprehensive response at the three levels of the organization) in January 2023.

https://bit.ly/3tH1RBT

## Dengue increased in 2023

There was a significant increase in the number, scale, and simultaneous occurrence of outbreaks of dengue in 2023, with the disease spreading into regions not previously affected.

According to a report published on 21 December 2023 over five million cases had been reported for the year with more than 5000 of the people infected dying from the disease.

Over 80 countries or territories reported cases in five WHO regions, with close to 80% of those cases (4.1 million) reported in the Region of the Americas. 

https://bit.ly/3HfiKXd

**Cover photo**

A child washes his face in front of his home in Tlogopakis village, Central Java province, Indonesia.

**Figure:**
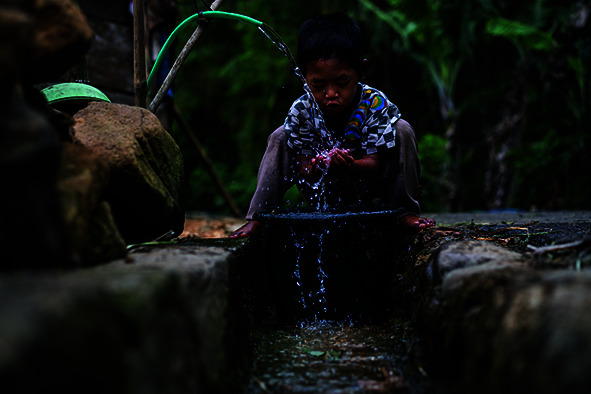
UNICEF / ljazah

## Cabo Verde certified malaria-free

WHO certified Cabo Verde as malaria-free. With the 12 January announcement, Cabo Verde joins the ranks of 43 countries and one territory to which WHO has awarded this certification.

Dr Matshidiso Moeti, WHO Regional Director for Africa called the achievement, “a beacon of hope for the African Region and beyond”, demonstrating what can be achieved with political will, effective policies, community engagement and multisectoral collaboration,

bit.ly/4aYtebl

## Preventing the uptake of e-cigarettes

There has been a sharp increase in the use of e-cigarettes among children and young people with rates exceeding adult use in many countries. This is according to a statement issued by WHO on 14 December, which notes that children aged 13–15 are using e-cigarettes at rates higher than adults in all WHO regions.

WHO called for urgent action is to control e-cigarettes to protect children, as well as non-smokers and to minimize health harms to the population.

https://bit.ly/3HeCA59

**Looking ahead**

6–8 February. 4th WHO Fair Pricing Forum. Virtual event. https://bit.ly/47w2Jab

27–29 February. Global high-level technical meeting on noncommunicable diseases in humanitarian settings. Copenhagen, Denmark. https://bit.ly/3vF2mwB

3 March. World Hearing Day 2024. Theme: changing mindsets. https://bit.ly/4aSdqa0

